# Developing lay summaries and thank you notes in paediatric pragmatic clinical trials

**DOI:** 10.1111/hex.13448

**Published:** 2022-03-04

**Authors:** Kanecia O. Zimmerman, Brian Perry, Emily Hanlen‐Rosado, Adora Nsonwu, Morgan D. Lane, Daniel K. Benjamin, Mara Becker, Amy Corneli, Best Pharmaceuticals for Children Act—Pediatric Trials Network Steering Committee

**Affiliations:** ^1^ Duke Clinical Research Institute, Duke University School of Medicine Durham North Carolina USA; ^2^ Department of Pediatrics Duke University Durham North Carolina USA; ^3^ Department of Population Health Sciences Duke University Durham North Carolina USA

**Keywords:** clinical trials, formative research, lay summaries, paediatrics

## Abstract

**Introduction:**

Better transparency of research results and participant engagement may help address poor participant accrual in paediatric clinical research. We conducted formative research to assess the acceptability of lay summaries and thank you notes, as well as to refine and expand guidance on participant and family engagement in Pediatric Trials Network's (PTN) pragmatic paediatric clinical research.

**Methods:**

Informed by draft PTN guidance, we conducted in‐depth qualitative interviews with adolescent clinical trial participants and caregivers of paediatric participants in four trials conducted by PTN across eight sites. Participants were shown multiple versions of mock lay summaries and thank you notes and asked questions on their preferences for content and layout, and on trial communications. We used applied thematic analysis to analyse the data.

**Results:**

We interviewed 27 individuals engaged in PTN research: 24 caregivers and 3 adolescents. During a trial, participants want regular updates on study progress, reminders of the study purpose and reassurances of data confidentiality. After the trial, participants want to learn the aggregated results, particularly medication effectiveness. Participants reported that lay summaries should include a review of the study's purpose, methods and length, and that they expect to learn individual‐level results. Participants stated that thank you notes must be of sufficient length to be meaningful.

**Conclusions:**

This is the first study to describe stakeholder preferences for thank you note content and layout. Using these findings, we finalized PTN's trial communication guidance for use in future PTN trials. Research is needed to determine the effect of lay summaries and thank you notes on improving public transparency regarding clinical trials and paediatric trial recruitment and completion.

**Patient or Public Contribution:**

By design, stakeholders (adolescent trial participants and caregivers of pediatric trial participants) contributed to PTN's guidance on the content and layout of lay summaries and thank you notes through their participation in the in‐depth interviews.

## 1 INTRODUCTION

Nearly 20% of paediatric randomized‐controlled trials are discontinued before completion, primarily due to poor participant accrual.^1^ Experts assert that better transparency and participant engagement may be key to improving participant recruitment and retention.^2^, ^3^, ^4^ Some existing data support this premise. In one study, >50% of parents of paediatric participants in a rheumatologic clinical trial reported increased interest in participating in subsequent research after receiving the results of the current trial in which their child was participating.^5^ In another study, mothers of children with and without cancer acknowledged interest in participating in subsequent epidemiologic research after the return of study results.^6^ Study participants have also expressed interest in learning study results even when there is no immediate or future personal benefit.^7^

According to the National Academy of Medicine (formerly The Institute of Medicine), return of results is a ‘matter of public transparency and respect’ for those who make scientific advances possible.^4^ Providing summaries of trial results to participants also presents an opportunity to avow participant and parental value, educate adolescent participants and their caregivers about clinical research and foster trust in clinical research as a whole. Despite recognizing the importance of engaging participants, 30% of paediatric clinical trials go unpublished, and >75% of trial participants are never informed of the trial's results.^1^, ^8^

Multiple reasons may account for the historical failure to return results to participants. Among these, return of individual results is particularly complicated, and unanswered questions remain regarding content, timing, methods, maintenance of privacy and related ethics.^9^, ^10^ Nevertheless, communication of aggregate results through lay summaries is likely a feasible and necessary endeavour for public health, for which investigators have evaluated the acceptability and impact.^11^, ^12^, ^13^ Prior studies have focused on lay summary distribution in the setting of life‐threatening illness or chronic disease, where traditional, randomized trials evaluate the safety and efficacy of targeted therapeutics.^5^, ^6^, ^11^, ^14^ Little is known about the provision of lay summaries outside these specific patient populations and study designs. In particular, investigators of pragmatic trial designs, where trials are occurring within the context of clinical care, have little guidance as to the potential benefit, as well as the appropriate content and method of distribution, for lay summaries. Because pragmatic trials are increasingly common and designed to decrease participant burden and improve recruitment and retention, we must answer key questions regarding return of aggregate results to study participants within this context. Additionally, evidence on participants' perspectives on receiving thank you notes within pragmatic paediatric clinical research is limited. As a model infrastructure for the design and implementation of pragmatic clinical trials in children, the Pediatric Trials Network (PTN) sought to fill these critical knowledge gaps.

We describe research that assessed the acceptability of lay summaries and thank you notes for purposes of refining and expanding PTN guidance on trial communications with participants in pragmatic paediatric clinical research studies.

## 2 METHODS

### 2.1 Development of PTN guidelines for lay summaries and thank you notes

Available evidence suggests that aggregate results should present unbiased, nonpromotional study summaries and results to study participants while taking into account health literacy principles, such as writing summaries at the sixth‐ to eighth‐grade reading level and using plain language.^15^ Providing obligatory thank you notes to research participants is also recommended;^15^ however, no guidance is currently available on writing thank you notes for trial participants and their families.

Considering the lay summary guiding principles, the PTN collaborated with science communication experts to develop its own guidance document for drafting lay summaries for pragmatic paediatric clinical research. According to the draft guidelines, PTN lay summaries should be written at an eighth‐grade reading level with the following characteristics: (1) descriptive headers; (2) bullet lists; (3) graphics to illustrate concepts and aid in comprehension; (4) a link to additional information on the study; and (5) answers to the following questions:

1. Why was this study needed?
2. What kind of study was this?
3. What happened during the study?
4. What were the study results?
5. What side effects did (infants, children, adolescents, etc.) experience?
6. What happens next?
7. Who conducted the study?
8. Where can I learn more about this clinical trial?

### 2.2 Development of mock lay summaries and thank you notes

Using PTN lay summary draft guidelines, we developed three mock variations of a single lay summary that described the results of a recently completed PTN study for use in the formative research. We created an unformatted version, and two different formatted examples with graphics and photographs (Supporting Information eAppendix). All three summaries contained the same content; only the layout differed. Without the existence of prior guidance for provision of thank you notes, we opted to test three different versions of thank you notes. We developed a minimal text and limited graphic version, as well as two examples with expanded text and photographs (Supporting Information eAppendix). Only the photograph varied in the expanded text examples.

### 2.3 Evaluating acceptability of lay summaries and thank you notes

#### 2.3.1 Study design

Using a qualitative descriptive study design,^16^ we conducted in‐depth, one‐on‐one interviews with adolescent participants of PTN studies and caregivers of paediatric PTN study participants. These individuals were currently or recently engaged in one of the four PTN studies described below. To conduct the formative research, PTN partnered with The BASE Lab in the Duke University Department of Population Health Sciences.^17^

#### 2.3.2 Study sites

Eight sites (Table 1) that were conducting at least one of the following four studies participated in the formative research: (1) Pharmacokinetics of Antiepileptics in Obese Children and Adolescents (AED; children 2 to <18 years of age, with obesity, receiving one of four antiepileptics per standard of care); (2) Pharmacokinetics of Understudied Drugs administered to Children Per Standard of Care (POPs; children 0 to <21 years of age, administered one of >20 drugs per standard of care); (3) Pharmacokinetics and Safety of Anesthetics and Analgesics in Children and Adolescents (ANA; children 2 to <18 years of age receiving anaesthetics and analgesics per standard of care); and (4) Antibiotic Safety in Infants with Complicated Intra‐abdominal Infections (SCAMP; premature infants randomized to specific antibiotic regimens or administered specified regimens per standard of care). We identified these studies because they represent the range of studies (including participant age, disease states and study designs) conducted by PTN to enable testing of the approach across a broad range of PTN studies. In addition, these studies enrolled participants within the prior 5‐year period, increasing the potential to contact participants and caregivers.

**Table 1:** Participant characteristics

Characteristic	No. (%) (*n* = 27)
Age	
14–17	3 (11.1)
18–24^a^	1 (3.7)
25–34	10 (37.0)
35–44	10 (37.0)
45–49	3 (11.1)
Gender	
Female	27 (100)
Race^b^	
White	14 (51.9)
Black or African American	8 (29.6)
American Indian or Alaska Native	2 (7.4)
Asian	2 (7.4)
Other^c^	4 (14.8)
Ethnicity	
Hispanic or Latino	5 (18.5)
Current grade in school—adolescents	(*n* = 3)
8th–10th grade	3 (100)
Education—caregivers	(*n* = 24)
Some high school (9th–12th grade)	1 (4.2)
High school diploma or equivalent	4 (16.7)
Some college credit	7 (29.1)
Associate degree	5 (20.8)
Bachelor's degree	6 (25.0)
Master's degree	1 (4.2)
Employment—caregivers	(*n* = 24)
Employed full‐time	9 (37.5)
A homemaker	8 (33.3)
Employed part‐time	4 (16.7)
Out of work and looking for work	1 (4.2)
A student	1 (4.2)
Unable to work	1 (4.2)
Location of affiliated PTN research sites	
Durham, NC	6 (22.2)
Little Rock, AR	5 (18.5)
Dallas, TX	4 (14.8)
Wilmington, NC	3 (11.1)
Jacksonville, FL (Site 1)	3 (11.1)
Chicago, IL	3 (11.1)
Portland, OR	2 (7.4)
Jacksonville, FL (Site 2)	1 (3.7)
Affiliated PTN study	
POP01^d^	13 (48.1)
AED01^e^	9 (33.3)
SCAMP/ABS01^f^	4 (14.8)
ANA01^g^	1 (3.7)

Abbreviations: AED01, Pharmacokinetics of Antiepileptics in Obese Children; ANA01, Pharmacokinetics and Safety of Anesthetics and Analgesics in Children and Adolescents; POP01, Pharmacokinetics of Understudied Drugs Administered to Children Per Standard of Care; PTN, Pediatric Trials Network; SCAMP, Antibiotic Safety in Infants with Complicated Intra‐abdominal Infections.

^a^ Includes only caregiver participants.

^b^ Participants selected all that applied.

^c^ Two participants indicated Hispanic.

^d^ Study design: Opportunistic; Therapeutic Area: Several; Intervention: Multidrug; Endpoint: pharmacokinetics.

^e^ Study design: Opportunistic; Therapeutic Area: Seizures; Intervention: Levetiracetam, Valproic acid, Topiramate, Oxcarbazepine; Endpoint: pharmacokinetics and safety.

^f^ Study design: Randomized; Therapeutic Area: Intra‐abdominal infection in infants (<3 months); Intervention: ampicillin, metronidazole, clindamycin, piperacillin‐tazobactam, gentamicin; Endpoint: Safety.

^g^ Study design: Opportunistic; Therapeutic Area: Analgesia and Anesthesia; Intervention: Hydromorphone and Ketamine; Endpoint: pharmacokinetics/pharmacodynamics and safety.

#### 2.3.3 Sample size and recruitment

We aimed to enrol 24 caregivers and 12 adolescents, so we could gather a range of participant perspectives across multiple PTN sites. PTN site investigators and study coordinators at the participating sites purposively^18^ identified and recruited current and past adolescent participants (aged 12–17) and caregivers from the studies described above. These individuals were recruited based on investigators' and coordinators' perceptions of individual interest in participating in an interview on trial communications (e.g., determined from previous interactions during PTN study implementation). Additionally, to ensure that the overall interview sample was as diverse as possible, we provided suggestions on which races and ethnicities to recruit based on the enrolled study populations at each site for selected PTN studies.

#### 2.3.4 Data collection

We mailed participants colour copies of all materials in advance of the interview, with a request not to review the materials until the interview. All participants received the same set of mock lay summaries designed for a single trial and thank you notes; participants were not engaged in the trial described in the summary. The interviews were conducted on the telephone by an interviewer who had no prior interactions with the study participants. Informed by the best practices for testing materials,^19^, ^20^, ^21^, ^22^ participants reviewed each version of the lay summary and thank you note during the interview, and answered questions on their preferences for lay summaries and thank you note content and layout. We also explored preferences for caregiver and participant communications engagement with PTN during and after PTN studies, such as frequency and delivery route of communications; expectations for individual‐level results; and perceptions of clinical trials in general and within PTN. Adolescent and caregivers were asked the same questions. All interviews were audio‐recorded with participants' permission.

#### 2.3.5 Data analysis

We used applied thematic analysis^23^ to analyse the data, following guidance on establishing validity in qualitative research.^24^ Using NVivo 12,^25^ three analysts applied structural codes^26^ to segment participants' narratives into broad conceptual categories (e.g., informational needs). Intercoder reliability checks were conducted at three separate time points during the structural coding process. Discrepancies in code application were resolved through analyst discussions, and codebooks were revised and previous transcripts were recoded as needed. After structural coding was completed, each structural code was assigned to two analysts to review the coded text in detail. The analysts then: (1) independently created a list of proposed content codes^26^ to identify and label the specific information that participants described within the conceptual categories (e.g., informational needs after study closure); (2) met to compare their proposed content codes for each structural code and agree upon a draft content codebook; (3) independently applied the draft content codes to the same text within a portion of the structural code to ensure that the data are fully captured within the proposed codebook; (4) met to review and discuss application of the content codes; and (5) made revisions to the codebook accordingly. Each structural code was then assigned to a single analyst to content‐code the remaining text using NVivo 12.^25^ After content coding was completed, the analysts organized the codes thematically depending on the relationships between codes. Data reduction tables were created to aid in the identification of the most salient perspectives, and analysts created memos to summarize participants' narratives on these perspectives. As the final stage, analysts wrote analytical reports combining memos with illustrative quotes.

Here, we focus on a subset of the results related to areas of PTN guidance on lay summaries and thank you notes where stakeholder preferences are critical: informational needs, content and layout preferences, communication expectations and method of information delivery. We also summarize participants' perceptions of PTN trial involvement. Details regarding participant perceptions on the perceived value of and anticipated responses to receiving thank you notes and lay summaries are described elsewhere.^27^

#### 2.3.6 Ethics

The Duke University Institutional Review Board reviewed and approved the study. Site institutional review boards reviewed the protocol if local policies required review for recruitment activities only. For the caregiver interviews, an information sheet describing the study was provided since informed consent was waived. For the interviews with adolescents, caregivers provided their parental permission and adolescents provided their oral informed assent.

## 3 RESULTS

### 3.1 Study population

We interviewed 24 caregivers and 3 adolescents from diverse racial and ethnic backgrounds, education levels, employment statuses and time since enrolment in a PTN study. Nearly half of those interviewed (13/27, 48%) were participants or caregivers in the POPs study (Table 1).

### 3.2 Key findings

#### 3.2.1 Participant informational needs during the trial

During a trial, participants said they want to be kept informed about general information about the study, such as reminders of the study purpose and study procedures, as well as reassurances of confidentiality, particularly how participants' data will be used. They wanted updates about the medication being tested, such as the known side effects and what to watch for in their children, as well as updates on the study's progress, such as any interim findings. Participants suggested that information could be provided periodically based on study milestones or on a set schedule (e.g., monthly). A caregiver described the reasons she would want updates:

To see if we're going down the right path. [To see] if there's something that might have come up in the interim, and [if] there's information that's provided that might change the course of what we're doing as far as his care.

Another caregiver said:

I would like to be informed of any kind of mile markers within the study, if there was something big.

#### 3.2.2 Participants' content and layout preferences for lay summaries of study findings

After a trial has completed, participants said they wanted to learn the overall aggregated study findings, particularly whether the medicine under investigation was effective. A caregiver explained why receiving the findings is important to her:

I would like to find out what their findings were, and if they actually came up with a better solution to control the disease itself… It's important to me because I feel like in a way, it's telling my child's story because during this process, he has gone through a lot.

Nearly all participants said that the most important information to include in a lay summary on the overall findings is a review of the study's purpose, methods and length, as well as a clear summary of the study's results. Some also commented that they wanted to obtain more information about the drug and its side effects, as well as how many participants enrolled and the number of samples collected. A suggestion for lay summary content by an adolescent highlights the importance of describing the final study population who enrolled—a topic that a few other participants also mentioned should be included:

I would like to learn how many people were in the study and if I'm the only one… Because when I was there I was like, ‘Am I the only one?’ I didn't know if they had other people to compare what they were doing on me, too.

Participants overwhelmingly preferred the formatted lay summaries, which included graphics and pictures, compared to the unformatted summary with no formatting, graphics or pictures. Many participants explained that the unformatted version of the lay summary was informative, but boring. Of the two formatted lay summaries, the majority of participants preferred example #1 (Supporting Information eAppendix). The main reason described for this preference was that participants appreciated that the information was presented in a logical flow, as it helped them to understand the content better. Participants stated that the boxed text sections in example #2 (Supporting Information eAppendix) interrupted the coherent flow of the summary, which made it difficult to know where to read next.

Participants' perspectives differed on whether summaries would be acceptable for adolescents, focusing on concerns about their comprehension. Some participants suggested that PTN provide children ages 12–14 an easier‐to‐read summary while noting that 15‐ to 17‐year‐olds could likely understand the same version written for adult caregivers.

#### 3.2.3 Participant content and layout preferences for thank you notes

Participants overwhelmingly preferred the expanded text and graphic thank you notes (expanded examples #1 and #2, Supporting Information eAppendix) compared to the minimal text and graphic thank you note. Participants stated that the minimal text and graphic thank you note was ‘generic’, ‘plain’, ‘boring’, ‘impersonal’ and ‘not long enough’ to truly express appreciation, and that the additional paragraph was meaningful. A caregiver explained:

It makes me feel special, actually… the second paragraph, it makes you feel that you actually were part of something important.

Another caregiver said:

I think [the additional text is] more personal. It kind of gives you a real thank you, a genuine thank you… I like it. It gives you a clear‐cut thank you. ‘We couldn't do it without you. You make a difference’, kind of thing. And that feels good.

Preferences varied on what pictures to include in the thank you note. Participants who preferred the family picture said they appreciated the acknowledgement that participation in PTN studies is a family effort and that the picture was racially diverse as it made it relatable. Participants who preferred the photograph of the babies said it relates to the type of study in which their child participated.

#### 3.2.4 Preferred method of information delivery

Receiving study updates by postal mail was the most preferred option described among participants, followed by email, in‐person summaries at the hospital or clinic, and a web site.

#### 3.2.5 Expectations for receiving individual‐level results

The majority of participants said that they expect to receive individual‐level results when provided a lay summary of study findings. Several noted their expectation to learn how the medication affected their child's health, information that could inform the child's future medical care and their child's laboratory results, while expressing interest in comparing their child's results to the study‐wide sample. A caregiver said:

I would want to know: what was the normal with other kids? How did he [my child] scale? How did he compare to other kids in the study? Was it around the same? That's what I would like to know.

If they did not receive individual‐level results, many participants thought they would react with negative feelings, and several stated that they would feel ‘let down’, have unanswered questions remaining and might question their future involvement in research. Several noted that these negative reactions could likely be mitigated by being informed early on that they will receive only aggregate information at the end of the study.

### 3.3 PTN and perceptions of clinical trial involvement

Participants' descriptions varied as to whether their perceptions of clinical trials were positive or negative before their involvement in their respective PTN trial. Those who described having initial positive feelings thought clinical trials were ‘necessary’ and ‘help people in the future’; those who initially had negative feelings believed that clinical trials treated participants as guinea‐pigs, often did ‘not have enough explanation’ and were a ‘waste of time’. Far fewer participants described having had negative feelings about clinical trials after participating in the PTN trial. A caregiver said:

I'd never realized how important these studies were until I needed to be a part of one.

When probed, participants overwhelmingly felt that PTN trials were a good use of government funding. Participants' comments emphasized the importance of clinical trials in finding new effective treatments and ensuring appropriate medication use for all populations. A caregiver said:

I feel like anything we can try to do to get more knowledge and to further the greater good. Like in this case, my [child]—we definitely rely on this type of information. It's vital for us. I think it's a good use [of government funding] to keep helping other people.

## 4 DISCUSSION

Our study of PTN clinical trial participant and caregiver perceptions about lay summaries and thank you notes largely affirms the importance of the return of results to meet the expectations of trial participants, as well as demonstrate transparency and respect for those who make research possible. Indeed, research has documented that more than 90% of trial participants have previously expressed interest in knowing the results of the clinical trial in which they participated.^28^ More specifically, because we enrolled participants from across four diverse PTN studies, we learned that the design of the study (i.e., randomized, opportunistic) and extent of participant involvement do not diminish the desire and expectations for information about trial specifics, including drugs studied, study design and results. We also provide the first evidence to support thank you note content, layout and distribution, and provide a rationale and details about participants' preference regarding lay summaries and thank you notes that will enable implementation in future PTN trials. Based on these findings, we updated our draft guidance.

Participants were very interested in learning how trial results affected their individual children, and many reported anticipating negative feelings if they did not receive individual results. This desire for individual results is consistent with a body of literature that focuses primarily on genetic research results. Among >1000 respondents to a survey about parental preferences for receiving research results, >80% wanted to receive all research results for themselves and for their children.^29^ Considering this strong desire, there remains the possibility that trial participants and their caregivers may not view lay summaries as complete enough, despite their necessity and perceived benefit. This idea is an important call to action for investigators, ethicists and others to more carefully consider how individual results might be relayed to participants. This must include the consideration of timing of results, under what circumstances results should be shared and any potential unintended consequences.^9^, ^30^

Notably, provision of lay summaries could have an individual effect. For example, providers can discuss individual results with trial participants and potentially change their chronically prescribed medications or dosing of these medications based on PTN results. Yet importantly, PTN's efforts with lay summaries are also not without potential consequences. For example, many physicians and the general public may not be aware of the extent of gaps in paediatric labelling for drugs that are commonly administered to children.^30^ Providing PTN trial results in lay summaries that identify optimal dosing regimens in support of labelling could result in parental and physician concerns about experimentation in clinical care.^31^ Similar challenges have been noted in describing adverse drug events within lay summaries.^32^ To mitigate these issues, specific education to academicians, clinicians and the public regarding the current gaps in paediatric drug development and associated consequences is likely needed.

We learned that participants had preferences for receipt of lay summaries in print at the clinic or hospital, via mail or email. These reports are consistent with prior findings in the literature. For example, parents of children who survived retinoblastoma and pregnant women receiving antibiotics during pregnancy preferred written study results.^33^, ^34^ However, some studies have suggested that the method of aggregate summary delivery depended on positive or negative results of the study. For example, in a study of 400 adolescents with cancer, nearly 60% wanted a letter, followed by a phone call or an email summary with positive or neutral study results. For negative results, participants preferred a phone call or personal visit.^12^ Each method of lay summary delivery has potential benefits and drawbacks for use within the context of PTN and other innovative or pragmatic trials. Planning for distribution of results must clearly be done in the early stages of trial design and budgeting, and will likely require additional resources in terms of finances and personnel.

Our findings should be interpreted within the context in which the data were gathered. Since participants were purposively sampled, as is standard in qualitative research, our findings represent the views of those interviewed; an alternative group of participants may have shared different information. Notably, all participants were female, which may have biased the results. Additionally, we did not reach our sample size aim for adolescents and the overall number of adolescents interviewed was low, due to recruitment difficulties. Nonetheless, the findings are very informative for creating a roadmap on providing lay summaries and thank you notes in future PTN trials.

In conclusion, lay summaries and thank you notes, crafted and distributed based on participant and caregiver preferences, may help improve public transparency regarding clinical trials. For PTN, these methods provide an important opportunity for education, and permit investigators to acknowledge participant and caregiver volunteerism^15^ that is essential to complete PTN's mission.

## CONFLICT OF INTERESTS

K. O. Z. receives support from the National Institutes of Health (National Institute of Child Health and Human Development (K23 HD091398, HHSN275201000003I), the US Food and Drug Administration (UG3/UH3 FD 006797), the Duke Clinical and Translational Science Award (KL2TR001115‐03) and industry for neonatal and paediatric drug development (www.dcri.duke.edu/research/coi.jsp). M. D. L. received research support from the Duke BIGGER Program. D. K. B. Jr. receives support from the National Institutes of Health (award 2K24HD058735‐10), the National Institute of Child Health and Human Development (HHSN275201000003I), the National Institute of Allergy and Infectious Diseases (HHSN272201500006I), the ECHO Program (1U2COD023375‐02) and the National Center for Advancing Translational Sciences (1U24TR001608‐03); he also receives research support from Cempra Pharmaceuticals (subaward to HHSO100201300009C) and industry for neonatal and paediatric drug development, www.dcri.duke.edu/research/coi.jsp; M. B. receives support from the National Institutes of Health (National Institute of Child Health and Human Development (R01 HD089928, HHSN275201800003I) and the National Center for Advancing Translational Sciences (5U24‐TR001608‐04 and 3U24‐TR001608‐04S1). A. C. receives support through the Food and Drug Administration through the cooperative agreement U18FD005292 and grant R18FD005292, the National Institutes of Health through the Centers for AIDS Research (P30 AI064518), the National Institute of Allergy and Infectious Diseases (U19AI144182) and the National Institute of Mental Health (R21MH116778); she also receives industry research support from UCB (UCB‐14). B. P. receives support from U18FD005292 and R18FD005292, P30 AI064518, U19AI144182 and R21MH116778. A. N. receives support from U18FD005292 and R18FD005292, P30 AI064518, R21MH116778 and UCB‐14. E. H.‐R. receives support from U18FD005292 and R18FD005292, P30 AI064518 and UCB‐14.

## Supporting information

Supporting information.

## Data Availability

Interview transcripts contain potentially identifiable information and therefore are not publicly available due to privacy or ethical restrictions. Codebooks used for data extraction and analysis are available from the corresponding author on reasonable request.
