# Supplementary material for: Developing lay summaries and thank you notes in paediatric pragmatic clinical trials
Source: Health Expect. 2022 Mar 4;25(3):1029–37. doi: 10.1111/hex.13448 (PMC9122399; doi:10.1111/hex.13448)

**eAppendix: Draft Lay Summaries and Thank You Notes**

1. **Unformatted Version of the Lay Summary**

**THE AMPICILLIN IN INFANTS STUDY**

**WHY WAS THIS STUDY NEEDED?**

Ampicillin, a medicine used to treat infections, is the most commonly used drug in the neonatal intensive care unit (NICU). At the same time, no one has ever figured out how the drug is processed in newborns. The goal of this study was to find out how newborns process ampicillin and determine the safest and most effective dose.

**WHAT KIND OF STUDY WAS THIS?**

The study enrolled 73 newborns at nine sites who were already taking ampicillin to treat an infection. It was an open-label study, meaning that both the researchers and families knew that the infants were taking the drug. After the dose of ampicillin was given, researchers checked the levels of ampicillin in the infants’ bodies over time to find out what dose was safest and most effective.

**WHAT HAPPENED DURING THE STUDY?**

Over a three-month time period, a total of 142 blood samples were taken from 73 newborns (about 2 samples per newborn). These samples were taken as part of the newborns’ regular medical care, so only a few extra needle sticks were needed.

**WHAT WERE THE STUDY RESULTS?**

Most of the infants responded best to a dose that was based on:

- Their age
- How far along in the pregnancy their mothers were when they were born

Based on these findings, researchers were able to suggest a dose of ampicillin that was both safe and effective for newborns.

**WHAT SIDE EFFECTS DID NEWBORNS HAVE?**

None of the newborns had any side effects from taking the medicine.

**WHAT HAPPENS NEXT?**

The results of this study will be sent to the U.S. Food and Drug Administration (FDA), a government agency that approves drugs and devices used to treat patients. Our findings may be used to change this medicine’s “label,” or the printed information that is included along with the drug. The findings will also be published in scientific journals. Both the label and publications can provide doctors with information to help them give the safest, most effective dose of this medicine to children.

**WHO CONDUCTED THE STUDY?**

The study was conducted by the Pediatric Trials Network (PTN), a group of more than 100 research sites around the world that are working to find the safest and most effective doses of commonly used medicines for infants and children. Children aren’t just little adults. Their bodies are growing and changing, meaning that they process medicines differently than adults do. The PTN works to make sure doctors and families have the information they need to give children the right dose: one that will get them well and keep them safe.

*The study was made possible with support from the Eunice Kennedy Shriver National Institute of Child Health and Human Development*.

**WHERE CAN I LEARN MORE ABOUT THIS CLINICAL TRIAL?**

A summary of the results can be found online at **pediatrictrials.org**. If you have additional questions, please speak with the doctor or staff at your study site.

** This summary was completed on [month/year]. Newer information since this summary was written may now exist. This summary includes only results from one single study. Other studies may find different results.*

1. **Formatted Versions of Lay Summaries**


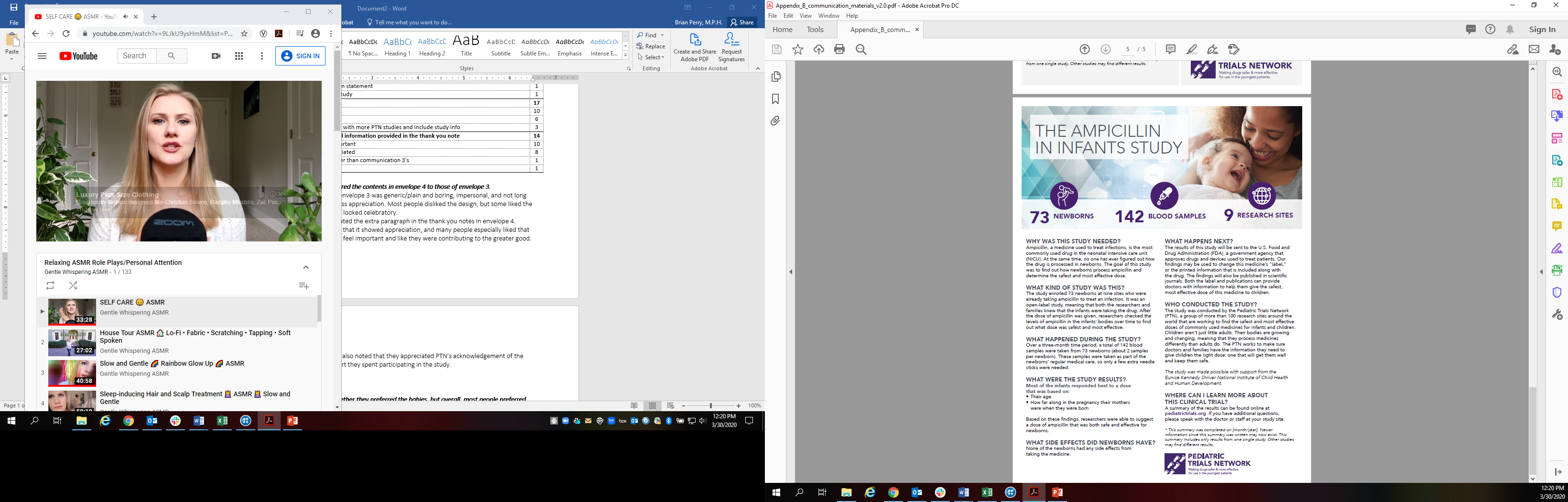


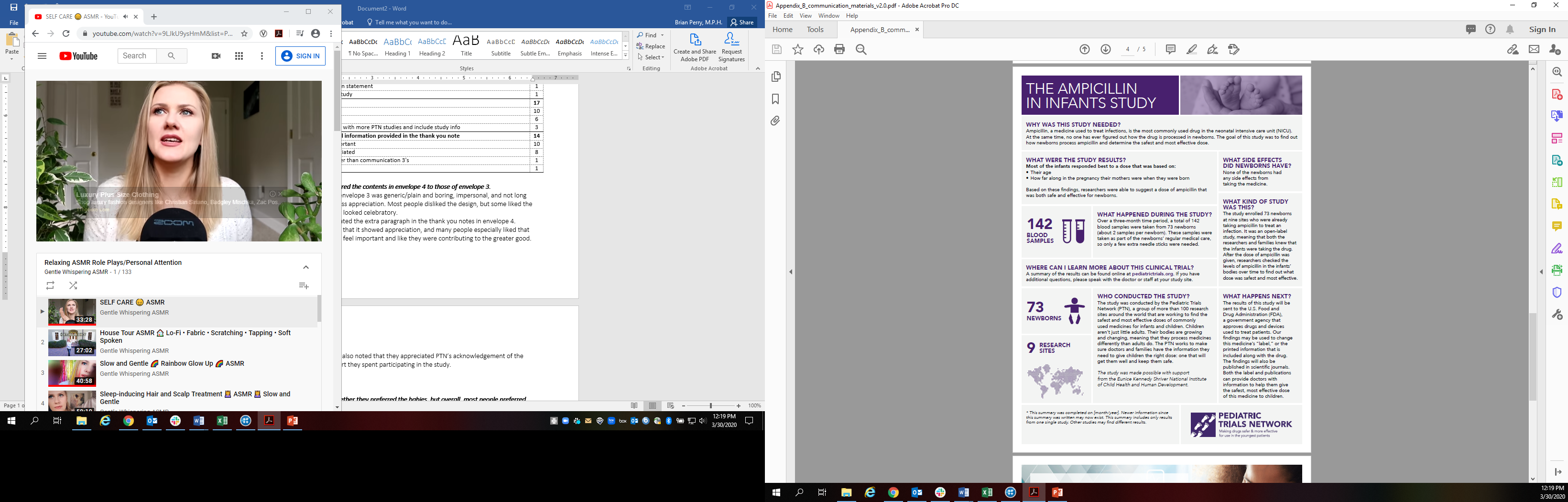


1. **Limited Version of the Thank You Note**


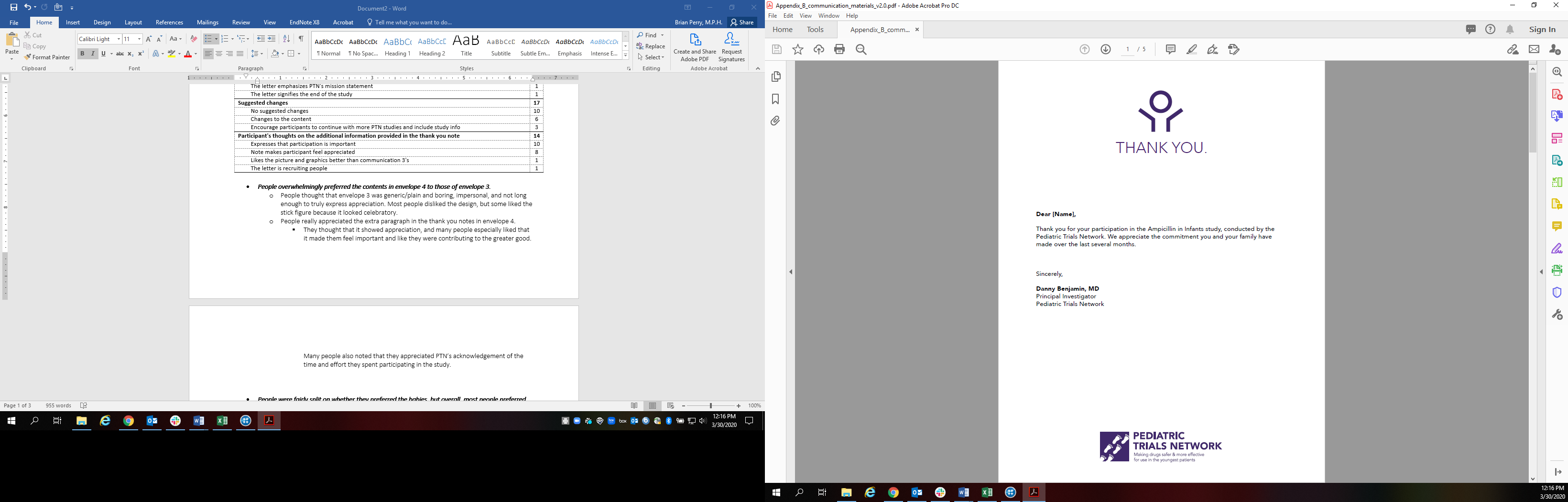


**2. Expanded Versions of the Thank You Notes**


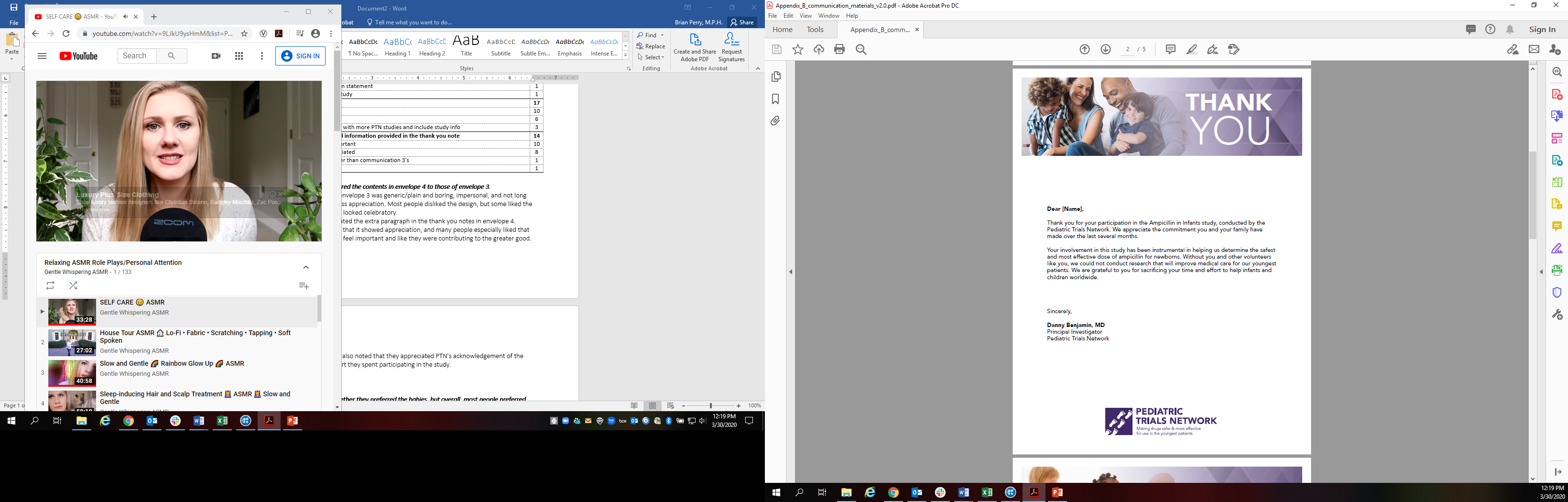


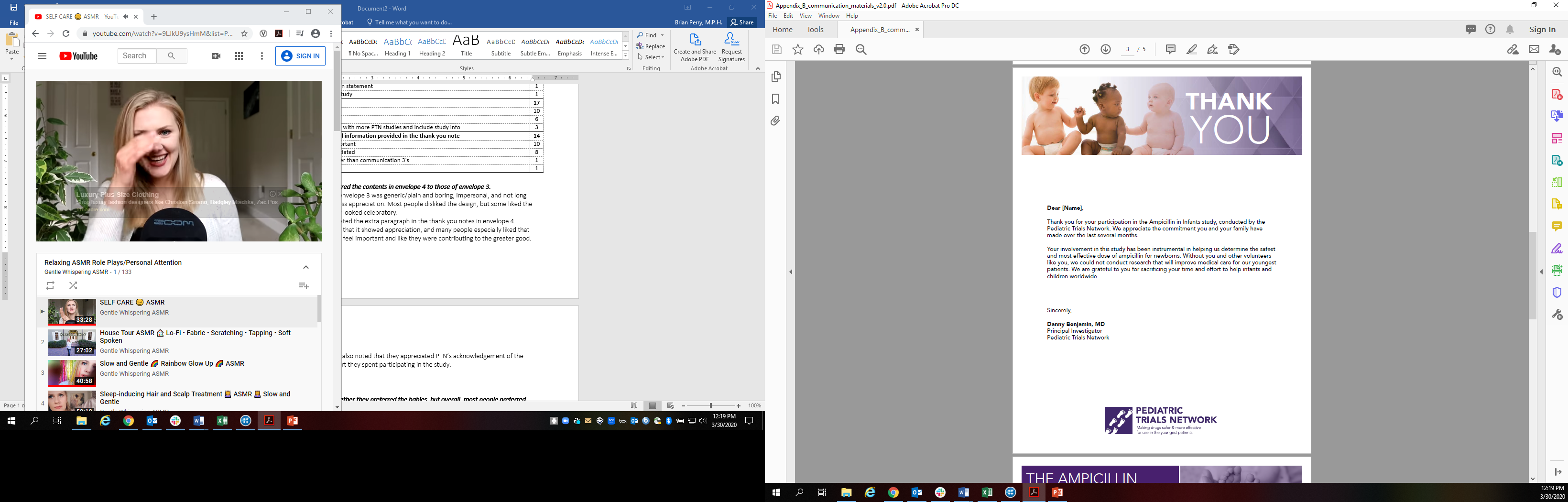

Supplement: Supplementary file 1 — Supporting information. [file HEX-25--s001.docx]
